# A New Figure of Merit for Organic Solar Cells with Transport-limited Photocurrents

**DOI:** 10.1038/srep24861

**Published:** 2016-04-26

**Authors:** Dieter Neher, Juliane Kniepert, Arik Elimelech, L. Jan Anton Koster

**Affiliations:** 1 Institute of Physics and Astronomy, University of Potsdam, Karl-Liebknecht-Str.24-25, D-14476 Potsdam-Golm, Germany; 2 Photophysics and Optoelectronics, Zernike Institute for Advanced Materials, Nijenborgh 4, NL-9747AG Groningen, The Netherlands

## Abstract

Compared to their inorganic counterparts, organic semiconductors suffer from relatively low charge carrier mobilities. Therefore, expressions derived for inorganic solar cells to correlate characteristic performance parameters to material properties are prone to fail when applied to organic devices. This is especially true for the classical Shockley-equation commonly used to describe current-voltage (*JV*)-curves, as it assumes a high electrical conductivity of the charge transporting material. Here, an analytical expression for the *JV*-curves of organic solar cells is derived based on a previously published analytical model. This expression, bearing a similar functional dependence as the Shockley-equation, delivers a new figure of merit α to express the balance between free charge recombination and extraction in low mobility photoactive materials. This figure of merit is shown to determine critical device parameters such as the apparent series resistance and the fill factor.

Due to intense research in material synthesis and device physics, record power conversion efficiencies of organic solar cells are now well exceeding 10%^1^,^2^. Conjugated organic materials, forming the active material of such cells, differ in many respects from their inorganic counterparts. Most importantly, because of their high absorption coefficient, organic semiconductors allow efficient photon harvesting for layer thicknesses well below 1 μm. On the other hand, their low charge carrier mobilities, typically below 10^−2^ cm^2^/Vs in bulk heterojunction blends, cause a considerable pile-up of photogenerated charge in the active organic layer, resulting in significant non-geminate recombination losses^3^. Organic solar cells, therefore, display non-ideal *JV*-curves with low fill factors. This asks for analytical approaches to treat the competition between charge extraction and recombination, and to relate characteristic device parameters such as the ideality factor or the fill factor to relevant material properties.

This goal is, however, aggravated by the fact that non-geminate recombination in organic solar cells is predominantly of second (or even higher) order^4^,^5^. Arnab and Kabir developed an analytical model taking into account drift, diffusion and trapping of photogenerated charge^6^. Good fits to experimental *JV*-curves were obtained for two popular polymer:fullerene blends, however under the assumption of a constant lifetime of electrons and holes in the active layer. This is at variance to a predominant second order recombination. Y.T. Set *et al.* derived a set of implicit equations taking into account second order recombination, which yielded *JV*-curves in good agreement to the results from 1D drift-diffusion (1D DD) simulations^7^. Ibrahim and coworkers provided explicit analytical expressions to describe the *JV*-curves of bulk heterojunction solar cells in the limit of uniform bimolecular recombination rates^8^. The recombination rate was written in terms of three empirical parameters which were then optimized to obtain the best fit to simulation results.

Our groups recently published two analytical approaches under explicit consideration of second order recombination. In the first paper^9^, by relating the current densities of recombination and extraction, *J*_rec_ and *J*_extr_, respectively, at short circuit conditions, the authors arrived at the dimensionless parameter


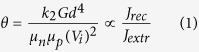


where *k*_2_ is the 2^nd^ order nongeminate recombination coefficient, *G* the generation rate, *d* the active layer thickness, μ_n_ and μ_p_ the electron and hole mobility, respectively, and *V*_i_ an internal bias (defined as the difference between the electrode work functions minus 0.4 V). Then, a large number of 1D DD simulations were carried out, with the physical parameters entering θ being varied over a wide range. Fill factors deduced from those simulated *JV*-curves displayed a unique dependence on θ, with little scatter, demonstrating the usefulness of this parameter to assess the quality of the *JV*-curves of organic solar cells. This important finding from simulation work was further supported by considering the performance of organic solar cells, made from blends of very different materials and compositions.

In a second paper^10^, 1D DD simulations were performed with parameters typical for organic solar cells. It was shown that the well-known Shockley-diode equation,


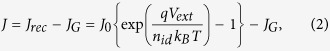


is inappropriate to describe the *JV*-curves of (low mobility) organic solar cells under illumination outside open circuit conditions. In Eq. 2, *J* is the total current density, *V*_ext_ the external bias, *J*_rec_ = *qdR* the non-geminate recombination current density, *J*_G_ = *qdG* the photogeneration current density, 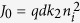 the dark generation (or dark recombination) current density, *q* the elementary charge, *k*_B_ Boltzmann’s constant, *T* the temperature and *n*_id_ the ideality factor (which is one for strict bimolecular recombination). To account for charge transport limitations, Würfel, Neher *et al.* suggested an analytical approach which relates *J* and *V*_ext_ to the Fermi-level splitting in the bulk of the solar cell under illumination:


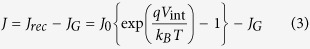



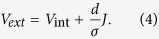


Here, *V*_int_ is the internal voltage describing the strength of non-geminate recombination. It is defined in a way that *qV*_int_ is the bulk quasi-Fermi level splitting, which, if being constant throughout the entire active layer, would cause the recombination current density *J*_*rec*_. Therefore, Eq. 3 is always valid. The transport properties of the organic semiconductor enter when relating the internal bias to the external bias via Eq. 4, where σ is the electrical conductivity given by


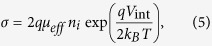


where 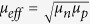 is the effective mobility and *n*_i_ the intrinsic carrier density.

To arrive at Eq. 4, the authors assumed the quasi-Fermi levels for electrons and holes to exhibit the same and constant tilt throughout the entire active volume. While this condition is automatically fulfilled at *V*_oc_ (in absence of surface recombination), the assumption was proven to be meaningful also at short circuit conditions through 1D DD simulations with balanced mobilities. Simulated *JV*-curves could be well reproduced with Eqs 3, 4, 5 for a wide mobility range.

Here, we show that these two approaches are related. We do this by deriving a closed form approximation of the *JV*-curves under transport-limited photocurrent conditions. We arrive at a new figure of merit, which relates characteristic parameters like the fill factor or the apparent series resistance of the device to the charge carrier mobilities, the active layer thickness and the bimolecular recombination coefficient.

The first step considers that at *V*_*ext*_ = *V*_oc_, also *V*_int_ = V_oc_. Rearranging Eq. 3 then leads to the well-known expression 
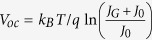
. This is reasonable because at open circuit the current density is zero and transport issues are irrelevant. Then with *J*_0_ ≪ *J*_G_ under realistic illumination conditions, Eq. 3 becomes:


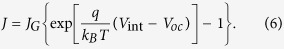


In this limit, the term 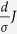 in Eq. 4 can be rewritten as


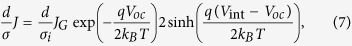


with 
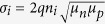
 being an effective intrinsic conductivity (see the Supporting Information for the derivation of Eq. 7).

To simplify Eq. 7 we consider that according to Eq. 6, most of the current change occurs in a rather small range of the internal bias around *V*_oc_. While *J* = 0 for *V*_int_ = *V*_oc_ by definition, it has attained 86% of the final saturation current already at 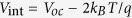. It is, therefore, reasonable to approximate the second factor in Eq. 7 with the use of 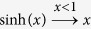:


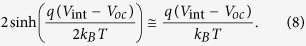


With the help of this approximation, Eq. 4 can be rewritten as


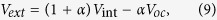


leading finally to


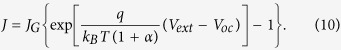


Equation 10 is an explicit analytical expression for the current-voltage characteristics of organic solar cells under the approximations outlined above. It is the central result of this publication. Here, α is a dimensionless parameter defined by


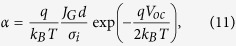


Finally, with 
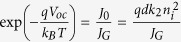
, the following equation relating α to the generation rate, the layer thickness and the relevant material properties is obtained:


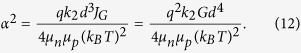


Comparing Eq. 12 with Eq. 1 finally yields a relation between α^2^ and the parameter θ in ref. 9:


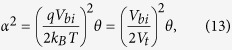


where 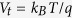 is the thermal voltage. Equation 13 shows that both approaches are, indeed, closely related, and that both can be used to assess the quality of a low mobility solar cell.

Before discussing in detail how the performance and in particular the FF of organic solar cells is set by the new parameter α, let us briefly consider the physical meaning of Eq. 9.

## Internal versus external voltage

By rewriting Eq. 9, we arrive at an expression relating *V*_int_ to *V*_ext_ and *V*_OC_:


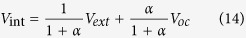


Note that *V*_int_ is a measure of the quasi-Fermi level splitting in the bulk while *V*_ext_ is the voltage applied between the two electrodes. Therefore, the difference between *V*_int_ and *V*_ext_ describes how the carrier population within the photoactive medium differs from the conditions set by the external bias. It is only for *V*_int_ = *V*_ext_ that the carrier density and with that the recombination rate in the bulk is strictly defined by the external bias, and that the Shockley-diode equation accurately describes the current-voltage curves of the solar cell. This ideal condition is, e.g. met if the quasi-Fermi levels are flat throughout the active layer and well-aligned to the work functions of the respective electrodes. However, a certain tilt of the quasi-Fermi level is always needed for moving photogenerated electrons and holes to the respective contacts, and this tilt will be small only for high electrical conductivities (high carrier densities and/or high mobilities). This condition is realized in doped crystalline inorganic semiconductors, where charge collection is mainly by diffusion. In contrast, for undoped low-mobility solids, a large driving force, given by the gradient of the quasi-Fermi levels, is needed throughout the bulk of the photoactive layer for efficient charge collection^11^. But then, *V*_int _≠ *V*_ext_ and the recombination current is not a sole function of *V*_ext_ (see Fig. S1). We refer to the situation when photogenerated charge carriers are extracted from all regions of the active layer, but requiring an appreciable gradient of the electron and hole quasi-Fermi levels for efficient collection as *transport-limited photocurrents*. This situation is distinctly different from the case of *space-charge limited photocurrents* (see the discussion in the following section). In that case, the build-up of space charge, due to imbalanced mobilities, doping or contact doping, causes the gradient of the quasi-Fermi level to disappear in part of the active layer. Because of low mobilities and, therefore, diffusivities, nearly all photogenerated charges recombine within these regions and no net photocurrent is extracted.

In the approximation described above, the internal voltage *V*_int_ is predicted to be a linear function of *V*_ext_ near *V*_oc_ (note that *V*_int_ = *V*_ext_ if *V*_ext_ = *V*_oc_). This is in accordance with the simulation results plotted in Fig. 1d in ref. 10. Importantly, the approximation predicts *V*_int_ = *V*_*ext*_ for *α* ≪ 1, which is the case if the active layer exhibits high electron and hole mobilities, a low recombination coefficient and a small layer thickness. Only then is the Shockley current equation for bimolecular recombination regained. In the other limit of very low mobilities (*α* ≫ 1), *V*_int_ is significantly larger than *V*_ext_ and fairly constant throughout a large bias range below *V*_oc_. It means that the inside of the solar cells is practically at open circuit conditions even for *V*_ext_ approaching short circuit. This is because slow extraction leads to a high carrier density in the bulk also at low external bias. Again, this finding nicely reproduces the simulation results in ref. 10. Consistent with these considerations, recent measurements on low mobility polymer:fullerene bulk heterojunction devices revealed a very weak drop of the carrier density when reducing the voltage from open circuit to short circuit conditions^3^,^12^. This emphasizes that for low mobility materials, where 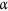 is considerably larger than one, *V*_int_ will significantly exceed *V*_ext_ for realistic solar cell driving conditions. Therefore, the Shockley equation is not applicable to the *JV*-curves of low mobility solar cells under illumination.

## JV-Curves

According to Eq. 10, our approximation for transport-limited photocurrents is similar to the Shockley equation, but with the term (1 + α) in the denominator of the exponent. As an important conclusion, this analytical approximation approaches the Shockley-limit when α goes to zero. Since the Shockley equation does not account for the transport properties of the active material (it assumes an infinitely high electrical conductivity), the value of α is a direct measure of the non-ideality of the device behavior due to insufficient charge extraction. Accordingly, 1 + α can be understood as an apparent ideality factor, which corrects the true ideality factor for transport losses. Note that this correction is not needed when deriving the ideality factor from the dependence of *V*_oc_ on light intensity, as the current at *V*_oc_ is always zero.

A lower mobility (larger α) will stretch the *JV*-curves along the voltage axis, around *V*_oc_, thereby weakening the dependence of the current density on the external bias, while maintaining its general (exponential) form. As a consequence, α will also affect the apparent series resistance calculated from the inverse slope of the *JV*-curve at *V*_oc_ according to:


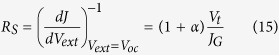


Large apparent series resistances are, therefore, an inherent property of low mobility solar cells. It is not possible to assess the quality of the contacts by simply calculating *R*_S_ from the slope of the *JV*-curve at *V*_oc_, without proper knowledge of the bulk charge transport properties^13^,^14^.

To evaluate the applicability of the analytical approximation outlined above, *JV*-curves calculated according to Eq. 10 were compared with the results of drift-diffusion simulations. These simulations were performed with similar parameters as in ref. 10, which are typical conditions for organic solar cell materials such as P3HT:PCBM or PCPDTBT:PCBM blends (see Table S1 for the used parameters). The injection barrier was set to 0.2 eV at both contacts (to avoid contact doping and band bending^15^,^16^), surface recombination was neglected (to avoid carriers leaving the device at the wrong contact^17^) and balanced mobilities were used (to avoid space charge effects)^18^. Though these conditions might appear arbitrary at first glance, they reflect the situation encountered in some state of the art solar cells, where a thin photovoltaic layer is sandwiched between two doped charge transport layers in a p-i-n-type fashion^19^,^20^ or where, because of a large active layer thickness, processes at the contacts are of minor importance^1^,^21^,^22^. Finally, only undoped active layers are considered here to avoid issues related to the formation of space charge zones^23^,^24^.

**Figure 1:**
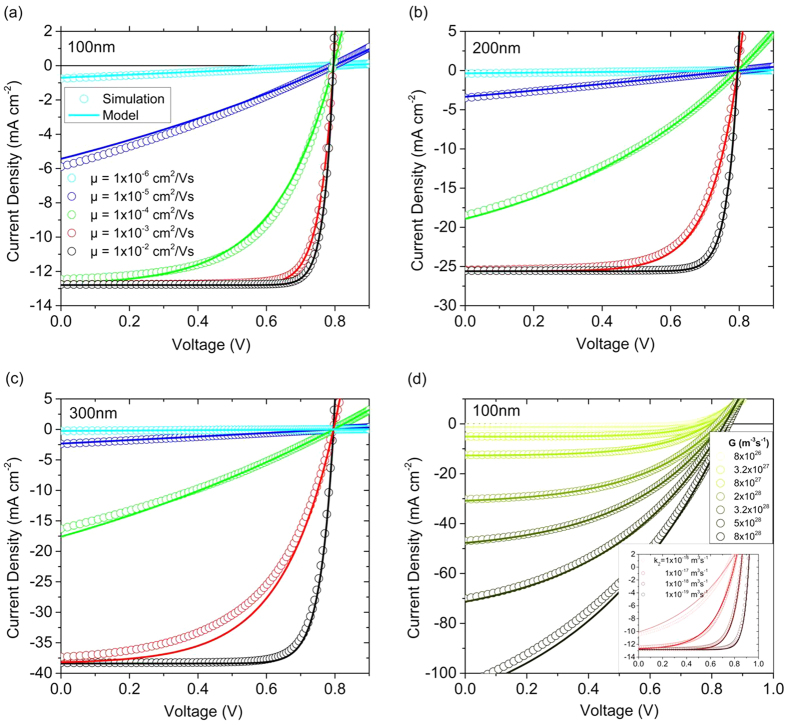
Comparison of *JV*-curves from 1D DD simulations (symbols) and the analytical approximation (lines) according toEq. 10. Simulations were performed with the parameters listed in Table S1 and the very same parameters were used to calculate the value of α according to Eq. 12. Only balanced mobilities are considered here. In Figure (**a**–**c**), the nongeminate recombination parameter *k*_2_ was set to 10^−11 ^cm^3^ s^−1^ and the generation rate *G* was 8 × 10^27^ m^−3^s^−1^, independent of free carrier mobility and active layer thickness. (**d**) displays simulations and calculations with *d* = 100 nm, μ_e_ = μ_h_ = of 10^−4^ cm^2^ V^−1^s^−1^ and *k*_2_ = 10^−11^ cm^3^ s^−1^, but now with *G* varied over two orders of magnitude (corresponding to illumination conditions between about 0.1 suns to 10 suns for a ca. 2 eV absorption bandgap absorber). The inset displays corresponding results for constant *G* but variable *k*_2_.

For these conditions, the analytical equation provides a very good description of the 1D DD simulation results for all mobilities and layer thicknesses tested here (Fig. 1a–c). This is an important finding given the fact that the mobility was varied from 10^−6 ^cm^2^/Vs to 0.01 cm^2^/Vs, corresponding to α ranging between 5.5 × 10^2^ and 5.5 × 10^−2^. Note that as simulations were performed with a constant generation rate (for simplicity), the photogenerated current density increases largely with increasing layer thickness. In fact, the analytical approximation is capable of describing the simulated results for a wide range of generation rates, see Fig. 1(d), proving its applicability to data gained at variable illumination conditions. The inset of Fig. 1(d) shows simulations with a constant generation rate but variable *k*_2_. Again, the analytical approximation provides a good description of the simulation results, though becoming less accurate for very large recombination coefficients.

As expected, conditions which lead to the build-up of space charge at the contacts (low injection barriers) or in the bulk (imbalanced mobilities) impair the accuracy of the analytical approximation (see SI for a detailed discussion). Theory and simulation work predict the photocurrent to become space charge limited if the photogenerated current approaches the unipolar space-charge limited current of the slower carrier^18^,^25^,^26^. In accordance to this, we find an only minor influence of mobility imbalance on the accuracy of the model for a 100 nm thick device, while significant discrepancies become obvious with increasing layer thickness. On the other hand, the influence of dark-injected charge due to low injection barriers is most obvious for a 100 nm layer thickness, but becomes less important with increasing layer thickness.

## Fill Factor and Figure of Merit

Having proven that the parameter α determines the shape of the *JV*-curves of low mobility solar cells, it is meaningful to search for an analytical expression relating the fill factor (FF) to the parameter α.

To arrive at such an analytical expression, we make use of the fact that Eq. 10 resembles the Shockley equation (except for the new term 1 + α in the denominator of the exponent). One commonly used expression to express FF as a function of *V*_oc_ is^27^:


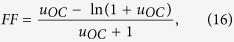


with the normalized open circuit voltage


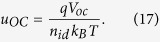


Unfortunately, Eq. 16 provides an accurate description of the true FF(*u*_oc_) dependence for high values of FF (and *u*_OC_), only (see Fig. S4). This is because in deriving Eq. 16, 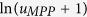 is replaced by ln(*u*_*OC*_ + 1) (which is a good approximation if the maximum power point, MPP, is close to *V*_oc_). Several improvements of Eq. 16 have been put forward, some with a different functional dependence of the argument of the logarithm on *u*_OC_, resulting in a considerable improvement the accuracy of the analytical expression for *u*_OC_ > 3 27. We found that the following empirical expression accurately describes the entire FF(*V*_oc_) dependence:


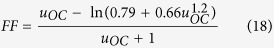


(see SI for the corresponding graphs). We propose Eq. 18, with *u*_oc_ written as


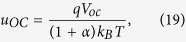


to provide a viable analytical description of the fill factor of solar cells with transport-limited photocurrents.

**Figure 2:**
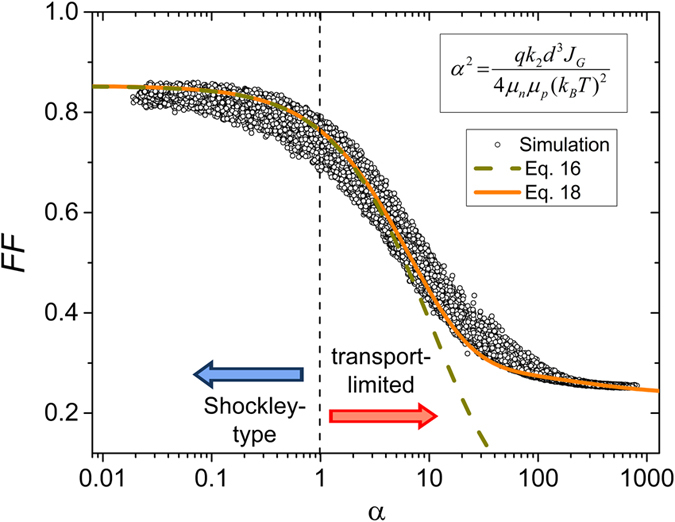
Fill factor (FF) as a function of the dimensionless parameter α. Open circles are FF-α points from simulated *JV*-curves with balanced mobilities and *V*_oc_ between 0.7 and 0.9 V (see ref. 9 for the simulation parameters). Dashed and solid lines show analytical FF-α dependencies according to Eqs 16 and 18, with the normalized open circuit voltage *u*_oc_ expressed as a function of α following Eq. 19 (*T* = 300 K, *V*_oc_ = 0.8 V). Photocurrents will become strongly transport-limited for α larger than 1, resulting in a progressive decrease of the fill factor when α increases beyond one.

To show the usefulness of this expression and of the parameter α to assess the quality of organic solar cells, we make use of the fact that α is related to the parameter θ according to Eq. 13. Figure 2 shows values of FF as extracted from the simulated *JV*-curves in ref. 9, but now as a function of α. Again, only simulations with balanced mobilities are considered here. Also, given the dependence of *u*_oc_ on the value of the open circuit voltage, data with *V*_oc_ below 0.7 V and above 0.9 V were omitted (which are actually rare cases for the conditions tested in ref. 9). Despite the fact that the simulations consider a wide range of mobilities, recombination coefficients and thicknesses, there is a surprisingly small scatter in the FF versus α plots. Also shown are the predictions of the analytical model, with *V*_oc_ set to an average value of 0.8 V. Evidentially, already the simple expression Eq. 16 captures the transition from the ideal behavior (α < 1) to the transport-limited regime (α > 1), which is the main topic of this work. As expected, Eq. 18 provides a much better description of the data, particularly when considering the fully transport-limited regime with high α’s and low fill factors. Note that Eq. 18 is by no means a direct fit to the data in Fig. 2. Rather than that it was developed to provide an accurate approximation of the FF(*u*_oc_) data retrieved from the Shockley-equation, and was then used in combination with Eq. 19 to yield a relation between FF and α for a given *V*_oc_ range.

As an important finding, the simulations and the analytical approximations reveal a rapid decrease of the FF for α > 1, when photocurrents become transport-limited. With Eq. 13, and *V*_i_ typically of 1.0–1.3 V, α = 1 translates into θ ≅ 10^−3^, which is the value at which FF is seen to become a strongly decaying function of θ in Fig. 3 of ref. 9.

## Discussion

The above considerations show that the *JV*-curves of solar cells with transport-limited photocurrents can be well described by an analytical equation containing the dimensionless parameter 1 + α. The *JV*-curves follow the well-known Shockley equation as long as α ≪ 1, while transport losses become important for α exceeding one. Important quantities such as the apparent series resistance and the fill factor can, therefore, be expressed as functions of α. We, therefore propose α as a new *Figure of Merit* for organic solar cells with transport-limited photocurrents. We note that an alternative transport figure of merit has been put forward in a recent publication by Stolterfoht *et al.*^28^. This paper considers that space charge formation and bimolecular recombination set in above a certain generation rate, limiting the amount of extractable charge. Our approach is different as it provides a proper approximation for the entire course of *JV*-curve in the application-relevant bias range, and as the figure of merit derived here contains all relevant parameters, including the active layer thickness.

Let us, finally, identify the conditions for which α < 1, meaning that the fill factor is safely above 70%. Since for such efficient devices, 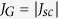, Eq. 12 can be rewritten as a function of well-measurable parameters. Then, the condition α < 1 translates into


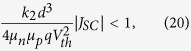


or


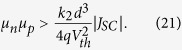


With *V*_th_ = 0.025 V at RT,


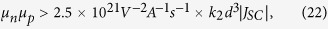


or in more practical units:


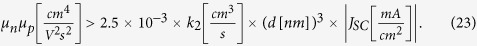


Efficient organic solar cells have short circuit currents of 15–20 mA/cm^2^. For such cells, values of *k*_2_ are typically 10^−11^ cm^3^/s^21^,^29^,^30^. Consequently, an effective mobility of at least 7 × 10^−4^ cm^2^/Vs is required for a 100 nm thick device while mobilities in excess of 4 × 10^−3^ cm^2^/Vs are needed to avoid significant transport losses in a 300 nm thick active layer. Such conditions have recently been realized through careful optimization of the blend morphology and polymer chain alignment^2^. Our considerations highlight the importance of high mobilities in order to realize high FFs in thick active layer devices.

Vice versa, Eq. 20 can be used to provide an upper limit for the active layer thickness for given values of the mobilities, non-geminate recombination coefficient and the short circuit current:


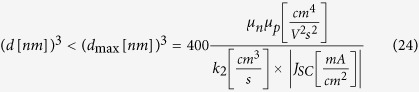


**Figure 3:**
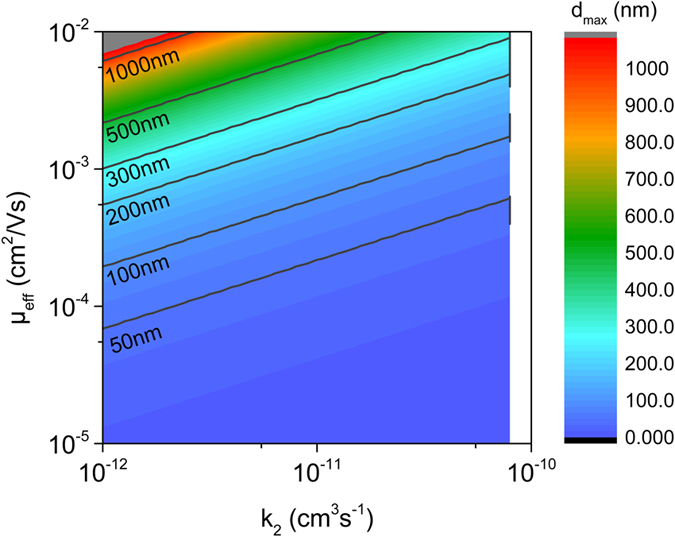
Maximum active layer thickness to avoid transport limitations. Shown is the maximum active layer thickness *d*_max_ calculated with Eq. 24 in dependence of the effective charge carrier mobility 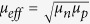 and the nongeminate recombination coefficient *k*_2_ for a short circuit current of 15 mA/cm^2^. Above *d*_max_, photocurrents become severely transport-limited.

To visualize how the effective mobility μ_eff_ and *k*_2_ affect the thickness at which transport limitations set in, *d*_max_ from Eq. 24 is plotted for a short circuit current of 15 mA/cm^2^ (Fig. 3). For a wide range of parameters, *d*_max_ is found of the order of 100 nm or lower. Particular conditions need to be met to prevent transport limited photocurrents for a thickness of 300 nm and above. All these predictions are in accordance with experimental findings. For example, for the high performance PTB7:PC_71_BM system [see ref. 30 for values], transport limitations are expected to set in at *d* = 70 nm, thus explaining the significant drop of the power conversion efficiency for an active layer thickness larger than 100 nm^31^. The situation is similar for DIO processed blends of the low bandgap polymer PCPDTBT with PCBM, where (with parameters documented in ref. 32), Eq. 24 predicts transport limitation to set in at ca. 50 nm, again agreeing to experimental findings^33^. On the other hand, with values typical for P3HT:PC_71_BM^34^, transport limited current behavior is predicted to become significant only for *d* > 250 nm. In perfect agreement to this, properly prepared blends exhibit high efficiencies for a wide range of active layer thicknesses^35^, and the thickness-dependent performance is in accordance with optical modeling results^36^.

We conclude that the analytical approximation derived in this paper provides a proper description of the *JV*-curves of low mobility solar cells under otherwise ideal conditions. We anticipate that large differences between experimental *JV*-curves and the analytical approximation will be indicative of conditions which are unwanted, such as a field-dependence of charge generation, space charge effects due to highly imbalanced mobilities or severe surface recombination.

## Additional Information

**How to cite this article**: Neher, D. *et al.* A New Figure of Merit for Organic Solar Cells with Transport-limited Photocurrents. *Sci. Rep.* **6**, 24861; doi: 10.1038/srep24861 (2016).

## Supplementary Material

Supplementary Information
