# Supplementary material for: A New Figure of Merit for Organic Solar Cells with Transport-limited Photocurrents
Source: Sci Rep. 2016 Apr 26;6:24861. doi: 10.1038/srep24861 (PMC4845057; doi:10.1038/srep24861)
Supplement: Supplementary Information [file srep24861-s1.pdf]

A New Figure of Merit for Organic Solar Cells with Transport-limited Photocurrents

Dieter Neher[1,*], Juliane Kniepert[1], Arik Elimelech[1], and L. Jan Anton Koster[2]

[1]Institute of Physics and Astronomy, University of Potsdam, Karl-Liebknecht-Str.24-25, D-14476 Potsdam-Golm, Germany

[2]Photophysics and Optoelectronics, Zernike Institute for Advanced Materials, Nijenborgh 4, NL-9747AG Groningen, The Netherlands

*corresponding author email address: neher@uni-potsdam.de

Supporting Information

Derivation of Equation 7

Using Equations 5 and 6, the term $\frac{d}{\sigma}J$ in Equation 4 can be rewritten as

$$d\frac{J}{\sigma} = d\frac{J_G}{\sigma_i}\exp\left(-\frac{qV_i}{2kT}\right)\left\{\exp\left(\frac{qV_i}{kT}\right)\exp\left(-\frac{qV_{OC}}{kT}\right) - 1\right\} \ , \quad \text{(S1)}$$

with $\sigma_i = 2en_i\mu_{\text{eff}}$. From this, simple arithmetic operations lead to:

$$\begin{aligned} d\frac{J}{\sigma} &= d\frac{J_G}{\sigma_i}\left\{\exp\left(\frac{qV_i}{2kT}\right)\exp\left(-\frac{qV_{OC}}{kT}\right) - \exp\left(-\frac{qV_i}{2kT}\right)\right\} \\ &= d\frac{J_G}{\sigma_i}\exp\left(-\frac{qV_{OC}}{2kT}\right)\left\{\exp\left(\frac{qV_i}{2kT}\right)\exp\left(-\frac{qV_{OC}}{2kT}\right) - \exp\left(-\frac{qV_i}{2kT}\right)\exp\left(+\frac{qV_{OC}}{2kT}\right)\right\}, \\ &= d\frac{J_G}{\sigma_i}\exp\left(-\frac{qV_{OC}}{2kT}\right)\left\{\exp\left(\frac{q(V_i-V_{OC})}{2kT}\right) - \exp\left(-\frac{q(V_i-V_{OC})}{2kT}\right)\right\} \end{aligned} \quad \text{(S2)}$$

finally leading to Equation 7:

$$d\frac{J}{\sigma} = d\frac{J_G}{\sigma_i}\exp\left(-\frac{qV_{OC}}{2kT}\right)2\sinh\left(\frac{q}{2kT}(V_i - V_{OC})\right). \quad \text{(7)}$$

Using the simplification $\sinh(x)\xrightarrow{x<1}x$ Equation 7 can be rewritten as:

$$d\frac{J}{\sigma} = d\frac{J_G}{\sigma_i}\exp\left(-\frac{qV_{OC}}{2kT}\right)\frac{q}{kT}(V_i - V_{OC}) = \alpha(V_i - V_{OC}), \quad \text{(S3)}$$

with $\alpha = \frac{q}{kT}\frac{J_G d}{\sigma_i}\exp\left(-\frac{qV_{OC}}{2kT}\right)$ as defined in equation (11) in the main document. Equation S3 is then inserted into Equation 4 to yield Equation 9 and finally Equation 10.

Transport-limited Photocurrents

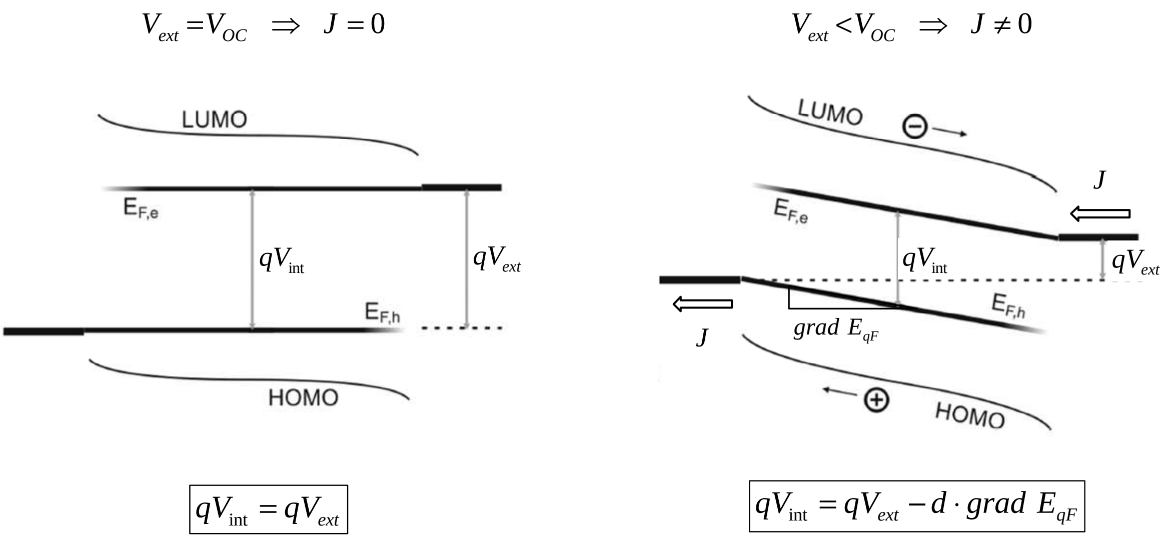

Figure S1: Simplified scheme of transport-limited photocurrents. At $V_{oc,}$ no current flows through the device and the quasi-Fermi levels of electrons and of holes in the bulk of the active layer are flat. Then, $qV_{int} = qV_{ext}$. However, for $V_{ext} < V_{oc}$ and low carrier mobilities the current of photogenerated charge is driven by an appreciable gradient of both quasi-Fermi levels throughout the entire active layer thickness, resulting in $V_{int} > V_{ext}$ (graphs adopted from Reference [1]).

1D Drift-Diffusion-Simulations

Simulations were performed with the program SIMsalabim which is described in detail in Reference [2].

Table S1: Parameters for 1D drift diffusion simulations. The following parameters were used in the numerical simulations in Figure 1, S2 and S3, if not stated otherwise.

Temperature T	300 K
Generation rate G	8 x 10^{27} $m^{-3}s^{-1}$ ('1 sun')
Thickness of active layer d	100 nm, 200 nm, 300 nm
Dielectric permittivity of active layer ε	3.4
Band gap energy E_{gap}	1.3 eV
Effective density of states N	5 x 10^{26} m^{-3}
Bimolecular recombination coefficient k_2	10^{-11} cm^3s^{-1}
Mobility of electrons and holes $\mu_{e,h}$	varies but μ_e=μ_h
Injection barrier E_B	0.2 eV

Imbalanced Mobilities

Shown in Figure S2 are numerical 1D DD simulations in comparison with the analytical model for a mobility ratio of 1:2 (a), 1:5 (b) and 1:10 (c) for a 100 nm layer thickness and a mobility ratio of 1:5 for a 200 nm layer thickness (d). Without loss of generality the electron mobility was set as the faster carrier mobility. For the 200 nm layer thickness, the generation rate was reduced to G=4x10^{27} m^{-3}s^{-1} to yield the same J_{SC} as for the 100 nm thick device.

The analytical approximation works quite well even for imbalanced mobilities, in particular for thin layers. For highly imbalanced mobilities and thick layers, the photocurrent will become space-charge limited and determined by the smaller mobility.[3, 4]

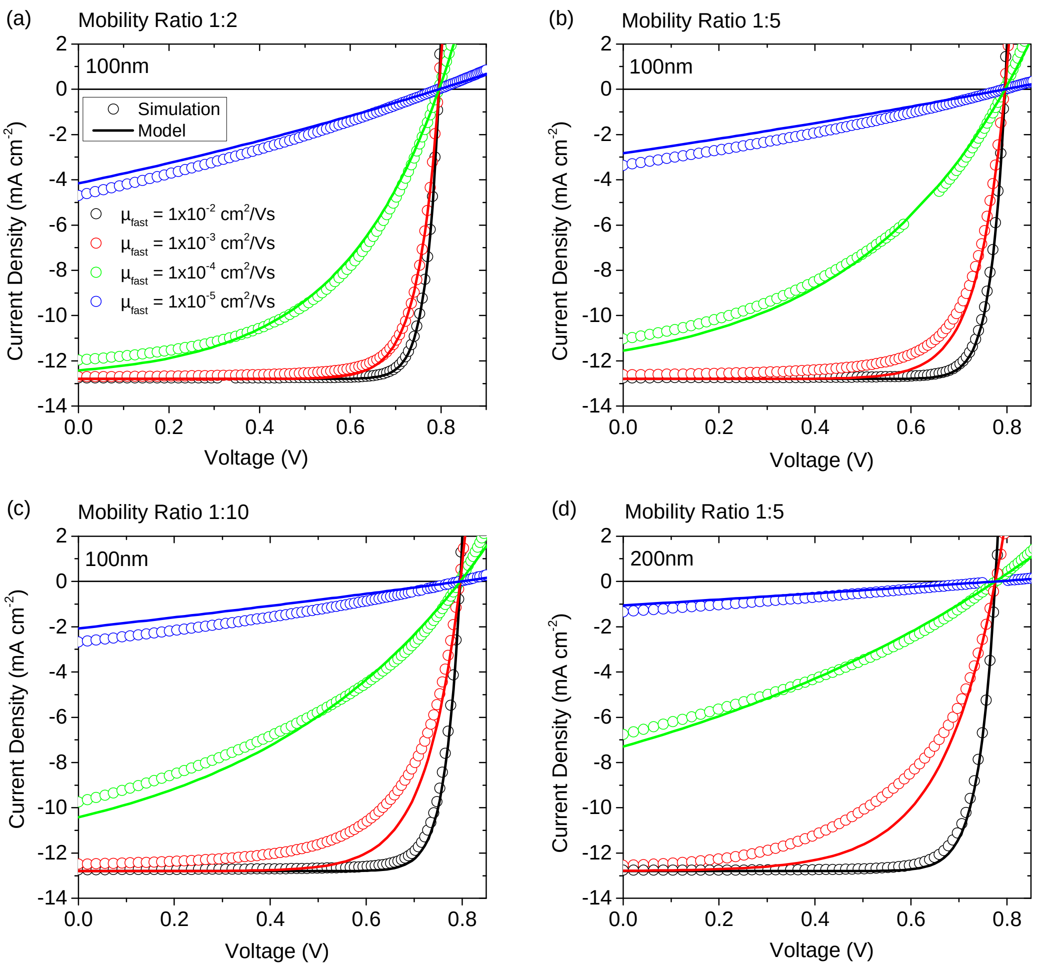

Figure S2: Simulated and calculated *JV*-curves in case of imbalanced mobilities. Symbols were calculated by 1D DD simulations with the parameters given in Table S1 and for different mobility ratio (with regard to the faster mobility). Lines show analytical *JV*-curves calculated with Eq. 10, with α calculated from Eq. 12.

Barrier Heights

Reducing the injection barrier heights causes efficient dark injection of majority carriers, accompanied by the formation of space charge layers at the contact. As a consequence, the electrical conductivity of electrons at the cathode (holes at the anode) will drastically increase, resulting in a flattening of the corresponding quasi-Fermi level near the charge injecting contact and an increased slope in the bulk of the active layer. As a consequence, charges will be more effectively extracted from the bulk, which is why the model tends to underestimates the photogenerated current density in certain regions of the *JV*-curves for thin active layers. As expected, the effect becomes almost irrelevant for thicker active layers.

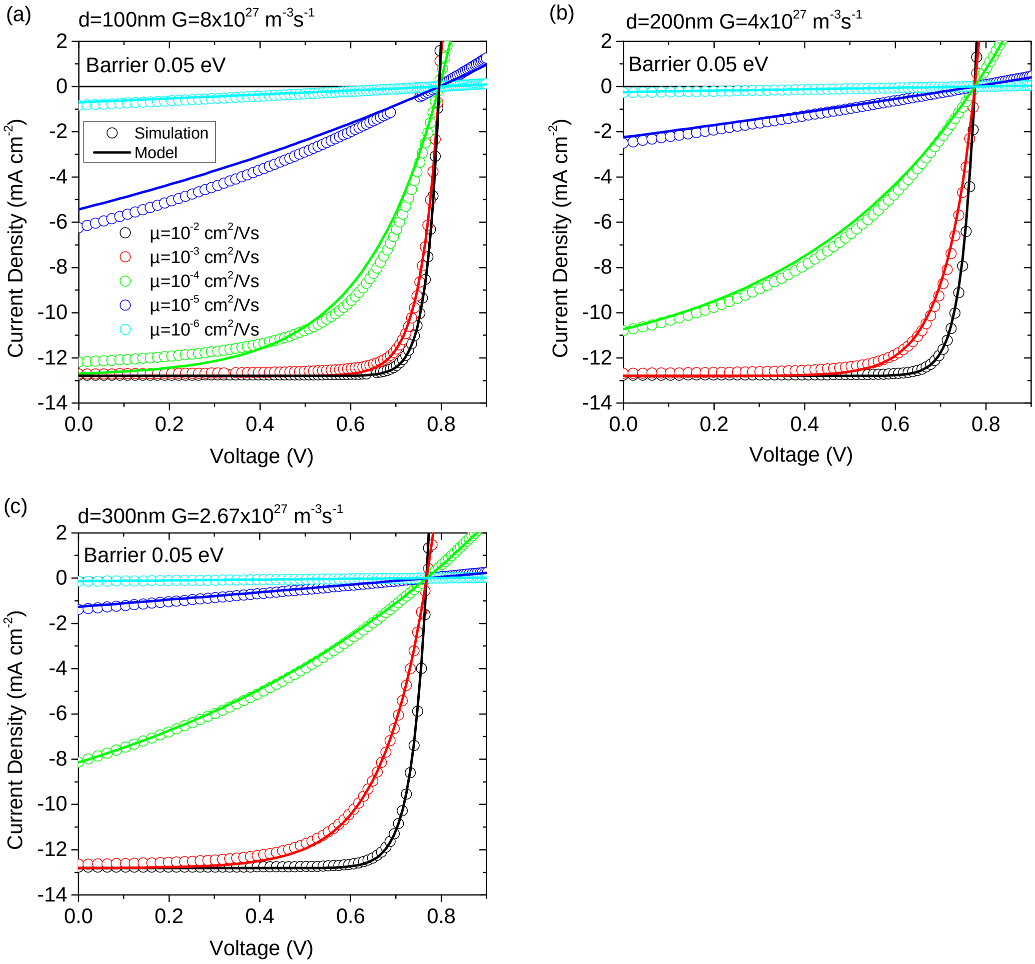

Figure S3: Simulated and calculated *JV*-curves for a reduced barrier height of 0.05 V. Symbols were calculated by 1D DD simulations and lines are analytical *JV*-curves according to Eq. 10 and Eq. 12. Only balanced mobilities are considered here.

Analytical $FF(u_{oc})$ Approximations

There is no explicit expression relating the fill factor to the normalized open circuit voltage. Therefore, a set of FF-u_{oc} points was numerically extracted from ideal Shockley-type JV-characteristics (Equation 1) for different J_G/J_0. These points are displayed as open symbols in Figure S4. Dashed lines show the well-known analytical expressions Equation 16 and Equation S4:

$$FF = \frac{u_{OC} - \ln(0.72 - u_{OC})}{u_{OC} + 1}, \qquad \text{(S4)}$$

which were developed to provide an accurate description of $FF(u_{oc})$ for high values of u_{oc} (and FF).[5] The solid line is the empirical relation Eq. 18, which was developed in this work to approximate the $FF(u_{oc})$ dependence over the entire FF range.

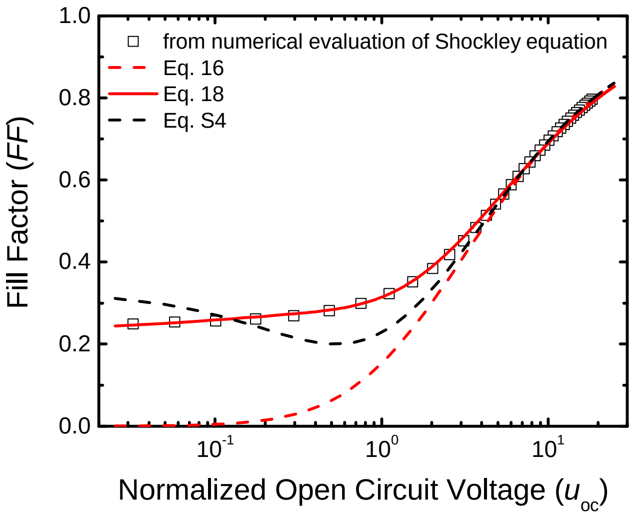

Figure S4: Fill factor FF as a function of the normalized open circuit voltage. Open symbols u_{oc} are FF-u_{oc} points, which were extracted from ideal Shockley-type JV-curves. The analytical approximations Eq. 16 and Eq. S4 (dashed lines) provide an accurate description for high values of u_{oc}, only. Good agreement over the entire FF-u_{oc} range is achieved with the empirical relation Eq. 18 (solid line).

References

[1] Albrecht, S.; Tumbleston, J. R.; Janietz, S.; Dumsch, I.; Allard, S.; Scherf, U.; Ade, H., Neher, D. Quantifying Charge Extraction in Organic Solar Cells: The Case of Fluorinated PCPDTBT. *J. Phys. Chem. Lett.* **2014,** *5*, 1131-1138.

[2] Koster, L. J. A.; Smits, E. C. P.; Mihailetchi, V. D., Blom, P. W. M. Device model for the operation of polymer/fullerene bulk heterojunction solar cells. *Phys. Rev. B* **2005,** *72*, 085205.

[3] Goodman, A. M., Rose, A. Double Extraction of Uniformly Generated Electron-Hole Pairs from Insulators with Noninjecting Contacts. *J. Appl. Phys.* **1971,** *42*, 2823-2830.

[4] Mihailetchi, V. D.; Wildeman, J., Blom, P. W. M. Space-Charge Limited Photocurrent. *Phys. Rev. Lett.* **2005,** *94*, 126602.

[5] Green, M. A. Accuracy of analytical expressions for solar cell fill factors. *Solar Cells* **1982,** *7*, 337-340.
